# Ethical Challenges in Organoid Use

**DOI:** 10.3390/biotech10030012

**Published:** 2021-06-28

**Authors:** Vasiliki Mollaki

**Affiliations:** Hellenic National Bioethics Commission, PC 10674 Athens, Greece; v.mollaki@bioethics.gr

**Keywords:** organoids, biobanking, ethics, bioethics, regulation

## Abstract

Organoids hold great promises for numerous applications in biomedicine and biotechnology. Despite its potential in science, organoid technology poses complex ethical challenges that may hinder any future benefits for patients and society. This study aims to analyze the multifaceted ethical issues raised by organoids and recommend measures that must be taken at various levels to ensure the ethical use and application of this technology. Organoid technology raises several serious ethics issues related to the source of stem cells for organoid creation, informed consent and privacy of cell donors, the moral and legal status of organoids, the potential acquisition of human “characteristics or qualities”, use of gene editing, creation of chimeras, organoid transplantation, commercialization and patentability, issues of equity in the resulting treatments, potential misuse and dual use issues and long-term storage in biobanks. Existing guidelines and regulatory frameworks that are applicable to organoids are also discussed. It is concluded that despite the serious ethical challenges posed by organoid use and biobanking, we have a moral obligation to support organoid research and ensure that we do not lose any of the potential benefits that organoids offer. In this direction, a four-step approach is recommended, which includes existing regulations and guidelines, special regulatory provisions that may be needed, public engagement and continuous monitoring of the rapid advancements in the field. This approach may help maximize the biomedical and social benefits of organoid technology and contribute to future governance models in organoid technology.

## 1. Introduction

Organoids are mini organs grown as 3D cell structures in the lab that display architectures and functionalities similar to in vivo organs, derived from Embryonic Stem Cells (ESCs), induced Pluripotent Stem Cells (iPSCs), adult stem cells and tissue-specific progenitors [1]. They have the ability to self-organize, they are multicellular and can be grown indefinitely. Multiple organoid systems have already been developed from both mouse and human stem cells, including taste bud organoids, salivary gland, esophagus, stomach, intestine, colon, liver, pancreatic, prostate, lung, retina, inner ear, kidney, heart, thyroid, skeletal muscle, bone, skin and brain organoids [2,3]. They can exhibit close resemblance to real organs, in terms of architecture and function, and therefore hold substantial opportunities for the investigation of complex human diseases, drug development, regenerative and precision medicine, as well as transplantation.

Despite the promises for science, the technology of organoids poses complex ethical challenges because it involves use of human tissues, production of sensitive personal data, long-term storage in biobanks, as well as the potential for some organoids to obtain human characteristics. Although to date there are no specific guidelines or regulations for organoid use and biobanking, there are several instances where organoids are already being used at the stage of clinical trials [4], demonstrating the quick pace at which this technology moves. As a result, considering the unique near-physiological characteristics of human organoids, a more thorough consideration of the ethical issues posed by organoids is necessary to achieve ethical use and societal acceptance of organoid technology.

This study aims to analyze the multifaceted ethical issues posed by organoids and to identify potential measures that need to be taken at various levels to ensure the ethical use and application of this technology. It is concluded that despite the multifaceted ethical challenges posed by organoid use and biobanking, we have a moral obligation to support and pursue organoid research, in order to make sure that we do not lose any of the potential benefits that organoids offer. A stepwise approach is recommended, which may help maximize the biomedical and social benefits of organoids and contribute to future governance models in organoid technology.

## 2. The Promises of Organoid Technology

One cannot deny that there are still certain limitations in organoid development and function than we need to overcome. For example, the lack of vascularization and maturation in the developing organoids, the lack of standardization in organoid establishment and quality control, the variability of phenotypes produced and the lack of inter-organ communication are remaining challenges [5]. However, organoid technology holds great potential in clinical translational research.

### 2.1. Alternatives for Drug Testing in Animals

First, organoids provide complementary approaches to the use of laboratory animals for scientific purposes. In vivo studies screening for novel drug compounds, testing efficacy and toxicity are necessary for drug approval by the competent authorities. Following proper validation as pre-screening systems for novel drugs, in vitro studies in organoids can substantially reduce the number of animals used [6]. Moreover, organoids provide greater experimental flexibility and accessibility compared to vertebrate animal models, allowing for extensive research at a lower cost.

### 2.2. Disease Modelling

Second, the ability of organoids to mimic human pathologies at the organ level will counteract the lack of appropriate animal disease models, particularly for chronic, infectious or complex diseases, and will facilitate the study of disease mechanisms. Even in cases where appropriate animal models exist, they cannot entirely reflect human physiology. Organoids can bridge this gap in research between animals and humans. They can be used in disease modelling aiming to develop advanced therapies for various human diseases. To name a few, they have already been used as models of genetic conditions such as cystic fibrosis [7], polycystic kidney disease [8] and Zika virus infection [9]. Brain organoids, in particular, have huge potential for modelling neurodevelopmental disorders, such as microcephaly [10], which are either impossible to model in animals or existing animal models are not appropriate.

### 2.3. Living Biobanks

Third, small tissue biopsies from humans can be used to develop human organoids which can be grown indefinitely. Derived either from healthy volunteers or patients, these organoids can be stored and serve as living biobanks for the study of different pathologies in translational research. Such biobanks do not only provide a source of biological material, but can also provide information on organ physiology and function. 

### 2.4. Precision Medicine

Fourth, human genetic variation may influence the disease onset, symptoms, severity, progression and drug response. Patient-derived organoids provide the means to develop personalized approaches and lead to precision medicine. They can be used to select for appropriate drugs in patients with genetic diseases or cancer, to predict response to drugs and choose better therapeutic options for each individual or groups of individuals. In other words, organoid biobanking can be a valuable resource to identify effective drugs against a broad spectrum of disease phenotypes. If these biobanks manage to cover the range of genetic variance in populations worldwide, they will eventually facilitate the design of powerful drug screening platforms, which will be effective for targeted groups of patients [11]. 

### 2.5. Regenerative Medicine

Fifth, organoids derived from healthy individuals can provide the basis for advanced therapies. Organoids comprise an exceptional source of stem cells for cell therapies and tissue engineering products with potential applications in numerous human diseases. A characteristic example is the transplantation of human embryonic stem cell-derived retinal tissue in two primate models of retinal degeneration [12]. Patient-derived organoids can even be combined with in vitro genome modification technologies, such as CRISPR (Clustered Regularly Interspaced Short Palindromic Repeats), to edit genetic mutations causing disease and replace existing pathological tissues. The study by Schwank et al. provided the proof-of-concept by using the CRISPR/Cas9 genome editing system to correct the Cystic Fibrosis Transmembrane Conductor Receptor (*CFTR*) locus by homologous recombination in cultured intestinal stem cells of cystic fibrosis patients, and the corrected allele is expressed and fully functional as measured in clonally expanded organoids [13].

### 2.6. Models of Organ Development

Sixth, the ability of organoids to self-organize and self-assemble makes these structures an excellent tool to model organ development, a process that cannot be studied in animal models due to interspecies differences [3].

### 2.7. Transplantation

Finally, organoids could provide an alternative source of organs for transplantation in humans. Although this application may seem remote and less realistic, at least for now, human organoids could potentially play a role in autologous, whole-organ replacement without having to face the challenges of immunocompetency and rejection. For example, the successful reconstitution of 3D nephric tubules and glomeruli, the two main components for kidney functions, from mouse and human PSCs provides insight on how organoid technology could be used in renal replacement strategies [14]. Again, transplantation applications of organoids could be combined with genome editing technologies to provide “healthy”, autologous organoids. For instance, the use of the CRISPR/Cas9 system in organoids to correct mutations in the *CFTR* gene causing cystic fibrosis, has also demonstrated that it is possible to use a similar strategy to generate autologous organoids for transplantation in patients [13].

## 3. Ethical Challenges in Organoid Use

Overall, organoids present with enormous potential for drug screening, disease modelling and therapeutic applications. However, their derivation and their current or future applications, raise a number of ethical issues that are discussed below. Some of the ethical dilemmas posed by organoids are similar to the ones raised by debatable issues existing for decades, such as research in human embryos and use of ESCs or informed consent and privacy of donors whose materials, e.g., cells, are used in existing technologies. Nevertheless, the use and storage of organoids pose additional, novel ethical challenges related to the potential acquisition of human “characteristics or qualities”, to their moral and legal status, to the level of acceptable organ maturation for certain applications, whether their creation constitutes life or whether they deserve special protection.

### 3.1. Source of Stem Cells

Organoids derive from fetal or adult tissues, from ESCs or iPSCs. ESCs are PSCs, possessing a nearly unlimited self-renewal capacity and developmental potential to differentiate into any cell type of the human body. This property allows ESC-derived organoids to serve as outstanding in vitro models for developmental biology. ESCs are isolated from the inner cell mass of in vitro fertilized blastocysts. Nonetheless, their isolation from human embryos, which deals with early forms of human life, creates significant ethical concerns over their use in research, including organoid research. Controversial beliefs can attribute a moral status to the human embryo ranging from that of human organs or tissues to that of a human being [15]. Consequently, the use of ESCs in organoid technology raises major ethical concerns on the value of human life and respect to human dignity.

Of course, this has also legal ramifications, as the human embryo is subject to stringent regulation in most jurisdictions. Under the “gradualist approach” adopted by several jurisdictions, the moral status of the embryo increases during its development as we move from fertilization, to implantation, to primitive streak and nervous system formation (the 14-day limit) and to subsequent developmental stages. Therefore, research on human embryos and consequent use of ESCs in organoids can be ethically acceptable depending on the developmental stage of the embryo, but always under strict conditions of informed consent and appropriate licensing. For example, research in embryos and human ESCs is prohibited in Italy and Germany, whereas the regulatory framework in Greece and Portugal allows for research in surplus embryos only until the 14th day of in vitro development, after informed consent of gamete donors and approval by the competent authorities. In only a few countries, such as the UK and more recently the Netherlands, the in vitro creation of embryos for research purposes is allowed after licensing [16].

Nevertheless, ESC use raises additional concerns over whether there is appropriate informed consent provided by the gamete donors or whether there is potential inducement. An informed consent for research purposes, which may include research for in vitro fertilization and infertility, ESC use, creation of ESC lines or use of ESCs for commercial purposes, may be considered too generic unless it is explicit enough to define the area of research and the potential uses of embryonic tissues. Therefore, a valid informed consent must be explicit enough to define the area of research and the potential uses of embryonic tissues. In the case where embryos are primarily created for research purposes, there are further issues that must be considered. These include health and safety risks for egg donors, as well as the compensation for egg donation, which remains a controversial issue, particularly because it entails the commodification of human body parts. 

The development of iPSCs provided a revolutionary alternative approach to the use of ESCs. Through the reprogramming of adult somatic cells, iPSCs exhibit pluripotency comparable to ESCs. Essentially, organoid technology was fired by studies showing that PSCs have the capacity to self-organize into the complex structure of an optic cup [17] and that intestinal organoids can be derived from single adult iPSCs [18]. Subsequent studies continued to demonstrate that adult stem cells can be propagated in various organoids, mimicking real organs. 

Although iPSCs may not be a complete alternative of ESCs in organoid technology, they can certainly help avoid the major ethical and legal challenges posed by the use of ESCs. iPSCs can circumvent the destruction of embryos, and set aside the significant issues of potential health risks and compensation for egg donors. They can be collected with minimally invasive or even non-invasive techniques, posing limited health risks to the donors, and most frequently, they are used for personal treatments of the donors themselves. In that sense, whenever science allows it, iPSCs may be preferable to ESCs in organoid technology.

### 3.2. Informed Consent of Cell Donors

The use of iPSCs in organoid technology raises less complex concerns compared to the use of ESCs, which relate mainly to the informed consent of tissue donors. Ethically, informed consent is the ultimate manifestation of respect of an individual’s autonomy. Legally, informed consent safeguards individuals’ or patients’ rights to autonomy and self-determination with diverse legal consequences in different jurisdictions. Nevertheless, whether donors are healthy individuals or patients, the purpose of donating cells for organoid creation can sometimes be unclear. Do they consent to the development of standard therapy? This presupposes that the end product (the therapy) has been previously validated and approved by competent authorities for this specific use. Do they consent to the development of an advanced therapy? This may include unproven therapies, for which limited or no proof on their safety and efficacy has been produced. Do they consent to research in organoid technology? If yes, do they consent to the development of disease models or novel therapies? This encompasses a systematic study which will lead to the documentation and establishment of results. Such questions are very difficult even for researchers to answer and the cell donors find it difficult to process all these possibilities and related information. In any case, a vague purpose of iPSC use for the development of organoids is not acceptable for a real and proper informed consent. The issue of informed consent is further discussed below in the context of organoid biobanking, which can include consent in both clinical and research settings.

### 3.3. Issues Specific to Embryoids

Studying the early phases of human development is of particular importance for birth defects and teratogenesis, as well as for prevention of implantation failure, pregnancy loss, infertility treatment and assisted reproduction. The main body of knowledge of embryonic development is derived from animal models, which, however, exhibit limitations due to morphological and genetic differences to humans. In vitro fertilization has enabled the study of human embryos, but this is restricted by the 14-day limit post-fertilization and poses serious concerns on the moral status of the embryo, as discussed above for the use of ESCs. Advances in stem cell biology and the use of embryonic and extraembryonic stem cells, including those derived from embryos, have made it feasible to study embryogenesis and embryo development in embryo-like structures called embryoids [19]. Unlike organoids that mimic a specific organ, embryoids model integrated development of the entire conceptus or a part of it, and may in the future have the potential of a full organism. Despite their differences, both organoids and embryoids show properties of self-organization and can be derived from pluripotent or differentiated cells [20]. In addition, they both show resemblance to their in vivo counterparts. Human embryoids exhibit similar morphological and gene expression features to real human embryos, which makes them the only resource to study embryo development beyond the limit of two weeks post-fertilization. In this context, embryoids are examined herein as distinct but similar 3D structures to organoids, which serve as models to investigate human biological processes or developmental diseases.

Although the use of human embryoids may help avoid the concerns of using human embryos, they provoke significant ethical controversy, mainly because some individuals may consider them a form of human life. The matter whether human embryoids could be considered as embryos holds implications for both research and policy. The moral status of these structures is debatable. They are derived from ESCs or iPSCs and they do not constitute zygotes derived from the fertilization of an egg with sperm. As a result, the narrow definition of a human being “from the moment of conception” that some individuals use may not be applicable here. Accordingly, the days post-fertilization cannot be defined and the 14-day limit may not be relevant, either. Hence, the question that arises is whether the 14-day rule could be breached, at least for embryoids derived from iPSCs. 

As a consequence, the legal status of these structures is also questionable. To date, there is little explicit regulation of human embryoid research. Depending on the definition of embryos in various jurisdictions, on the occasions that such a definition exists in the national laws, the use of embryoids in research can fall under existing provisions [21]. Hyun et al. make a distinction of different embryo models, depending on whether they attempt to model the integrated development of the entire conceptus, i.e., whether they have the potential to form a full organism or not, which may prove to be useful in future regulation of embryo models [22].

Another important issue to be considered here is that human embryoids derived from iPSCs could be considered by some as cloned embryos, as they are genetically identical to the cell donors, which could be subsequently used for therapeutic or reproductive applications. This will certainly complicate the regulation of embryoid use taking into consideration that worldwide policies on human cloning vary significantly, from permissive to restrictive or a complete lack of a specific policy. This is another reason why it is extremely important to provide a definition of an embryo and distinguish embryo models based on whether they have the potential to form a full organism or not. An equally important issue that is worth consideration is that of human cloning combined with eugenics. In pursuit of “perfection”, cloned human embryoids derived from iPSCs genetically modified to carry desired characteristics (physical or cognitive) may be considered by some as morally objectionable, leading to fundamental social inequalities and loss of inter-individual variability. 

As technology progresses, cell culture methodologies will be refined and the development of embryoids will better resemble the morphology and development of their in vivo counterparts. At the same time, this will elevate ethical concerns over the conduct of research in embryoids having the full potential to form an organism, and special legal oversight will be necessary. The degree of maturation is particularly relevant for embryoids. To which extend should human embryoids be allowed to mature? The answer to this question will have significant implications on their moral status, the degree of protection that they deserve and the “rights” of embryoids. The more they mature, the more closely they resemble human embryos and this implies that more research restrictions may be applicable. Rules, such as the 14-day limit or the appearance of a primitive streak, may also be applied in the case of such embryoids. The transfer of human embryoids to the uterus (either human or other mammalian) raises even more ethical concerns and may violate existing recommendations to ban human cloning.

### 3.4. Issues Specific to Brain Organoids

Human brain development and diseases affecting the brain are difficult to study in animal models, mainly due to differences in complexity, physiology and mechanisms between human and other species. In addition, based on moral grounds, the study of human brain in fetuses remains controversial. For the above-mentioned reasons, cerebral or brain organoids are extremely useful to investigate the complex processes of the brain. Various cerebral organoids have already been developed including forebrain, midbrain, hypothalamic and whole-brain organoids [10] exhibiting variable resemblance to their in vivo counterparts.

The main concerns on brain organoids revolve around the fact that these miniature organs constitute neural entities of human origin and whether they could obtain human characteristics, cognitive abilities or be sentient. Although researchers working on brain organoids may not directly aim to develop sentient organoids or organoids with cognitive abilities, this could be a consequence of their original aim to investigate human diseases and develop therapies. Thus, a key question that arises is whether they can exhibit consciousness, feel pain, respond to stimuli or even gain experiences in any way. The possibility that human brain organoids may develop consciousness has major complications. Of course, considering the lack of consensus on what constitutes consciousness, the lack of knowledge and the technical challenges on how to detect consciousness or investigate whether organoids can feel pain, it becomes evident that these issues are difficult to address. Some argue that the evaluation of the possible state of consciousness in brain organoids depends on the theory of consciousness that is adopted [23], while others support that existing tests to assess consciousness in brain-injured non-communicating patients may provide methods to assess consciousness in brain organoids [24]. In any case, the ability of brain organoids to host consciousness or feel pain depends on the degree of development and the maturity at different developmental states. 

A portion of researchers argue that scientific knowledge in brain organoids has not yet enabled organoids to interact and respond to stimuli or gain experience, and perhaps such concerns seem premature at present. Nonetheless, future advancements in methodology may allow brain organoids to develop cognitive functions, comparable to the human brain. Already, Muotri and colleagues have developed human cortical organoids, a brain region that controls cognition and interprets sensory information. These cortical organoids exhibited electrical activity, similar to the ones observed in premature babies born at 25–39 weeks post-conception [25]. Although brain organoids may not be mature enough to closely resemble the adult brain, their potential to host cognitive abilities demands strict ethical scrutiny before the technology progresses up to that point. 

To date, the degree of maturity that can be eventually reached by a brain organoid remains unknown and this has major implications on the informed consent provided by the cell donors. Uncertainties about the state of consciousness in brain organoids and whether they are able to feel can dispute that informed consent is really true and informed. To stretch this point, could there be any kind of connection between the cell donor and the brain organoid? How could such an issue be reflected in an informed consent? 

Whether and to what degree brain organoids can exhibit human characteristics has major implications on the moral status attributed to brain organoids. In the case that brain organoids are eventually found to exhibit even a minimal state of consciousness or found to be the least sentient, they may require special protection. This implies that limitations should be introduced to regulate the relevant research, including their storage, manipulation and destruction. For instance, if in the future it is demonstrated that brain organoids feel pain, then the comparison to animal studies is inevitable, and it will be necessary to impose rules equivalent to the principles of Replacement, Reduction and Refinement (3Rs).

### 3.5. Issues Specific to Gonadal Organoids

Establishing and characterizing testis and ovarian organoids from human iPSCs is a promising tool in male and female reproductive biology, pathology and toxicology. Indeed, studies have generated testis-like cells [26] and ovaries [27] with the ability to be cultured as an organoid from human iPSCs. Gonadal organoids offer an alternative to experiments that cannot be performed in humans due to ethical or regulatory issues, but their development and use certainly raise novel ethical concerns. 

As with other types of organoids, gonadal organoids can serve as a source of cells which could be likely used for in vitro fertilization (IVF). This includes cases where no viable oocytes can be extracted for IVF or cases of cancer where prepubescent girls undergo chemotherapy treatments destroying their oocytes. Although more research is necessary to reach the point that iPSCs can be used to develop gonadal organoids that could provide viable oocytes or sperm, progress in this field may open up new possibilities for infertility in the future. Indeed, this could help overcome the ethical issue of maternity and paternity in cases where infertile people use donated gametes that are genetically different from them. Even so, more complex ethical concerns are raised by the use of gonadal organoids in fertilization. In theory, a gonadal organoid developed by male iPSCs may be used to generate oocytes and vice versa, totally challenging the established religious beliefs or social standards that human reproduction requires a male and a female partner or donor.

The potential of gonadal organoids to be used for reproductive purposes also requires the explicit consent of tissue donors. In analogy to posthumous gamete and embryo use for reproductive purposes, which in many jurisdictions is permitted when written documentation from the deceased allowing the procedure is available, the use of gonadal organoids could be ethically acceptable only in the case that the cell donor has consented to this specific purpose. This issue is extremely sensitive considering that the original consent for the development of the organoid may have been obtained for other purposes, such as research or treatment, not explicitly for reproductive purposes. Informed consent by the cell donor in this case is a moral and legal recognition of the person’s autonomy and will certainly require regulatory oversight. 

In any case, a consensus should be reached at an international level on whether gonadal organoids could be used for reproductive purposes or whether this should be prohibited. It is important however, to define the scope of such a prohibition. The use of gametes originating form gonadal organoids may be banned for clinical use, i.e., transfer to the uterus after fertilization, but it could be allowed for research purposes to investigate infertility. 

### 3.6. Issues Specific to Multi-Organoid Complexes

The field of organoid research is undeniably advancing, and although not completely mature, scientific and technological developments may allow for connection of multiple human organoids to create multi-organoid complexes. With the advantage of the organ-on-a-chip technology and the use of microfluidics, assembling different organoids to multi-organoid complexes has already been demonstrated. For instance, merging organoid and organ-on-a-chip technology successfully generated complex multi-layer tissue models in a human retina-on-a-chip platform [28]. Skardal et al. also described a three-tissue organ-on-a-chip system, comprised of liver, heart and lung using bioengineered tissue organoids and tissue constructs that are integrated in a closed circulatory perfusion system [29]. Even more importantly, Xiang and colleagues recently established the fusion of two distinct region-specific organoids representing the developing thalamus or cortex, which are critically involved in sensory-motor processing, attention and arousal, and exhibited the feasibility of fusion of disparate regionally specific human brain organoids [30].

Although multi-organoid complexes broaden the horizons for drug testing, drug discovery and personalized medicine [31], these humanized models raise additional concerns and demand moral consideration. As Munsie et al. argue, “the degree of integrated biological functioning in multi-organoid complexes might trigger moral reactions on the appropriateness of creating and experimenting with such familiar, biologically humanized entities” [32]. As demonstrated by Xiang et al., this is of particular importance in cases where a brain organoid is connected with other nerve tissues or in cases where a brain organoid is connected with other organoids [30]. 

The potentiality of such human organoid complexes to accept and respond to stimuli or to exhibit some kind of autonomous behavior may provoke strong opinions on their human-like moral status, demanding special protection from harm. Consequently, the comparison between using multi-organoid complexes and animals is inevitable here. Multi-organoid complexes that include brain organoids would demand the obligation of researchers to seek alternative methods of experimentation. At least, they would demand the imposition of strict rules for pain minimization, manipulation refinement and appropriate methods of destruction or “sacrifice”, just as in animal studies, which would be assessed through in-depth ethics review processes by Research Ethics Committees. 

### 3.7. Gene Editing

Human organoid technology can be used in combination with genome (or gene) editing technologies to either study human diseases or develop novel therapies. Gene editing techniques can be applied to edit genes in ESCs, iPSCs, germ cells, somatic cells or even human embryos and hold great therapeutic potential. The CRISPR system has gained more interest compared to other technologies such as transcription activator-like effector nucleases (TALENs) and zinc-finger nucleases (ZFNs), because it is simpler, more flexible and has a low cost. The proof-of-concept study demonstrated that the CRISPR/Cas9 genome editing system can be used to correct a mutation in the *CFTR* gene in cultured intestinal stem cells of cystic fibrosis patients, and the corrected allele is expressed and fully functional as measured in clonally expanded organoids [13]. This study demonstrated the potential of CRISPR technology combined with patient-derived organoids and their utility as platforms for in vitro research and diagnostics. Since then, new applications of the CRISPR technology in organoids have appeared. CRISPR has been utilized in gut organoids to model cancer and hereditary diseases, in liver, pancreatic or mammary organoids to model cancer and in kidney organoids to model polycystic kidney disease (reviewed in [33,34]).

Furthermore, genome editing technologies offer a significant advantage in representing rare genotypes in organoid development. Donors exhibiting unique or rare genotypes may be extremely valuable in organoid technology, but this creates an enormous ethical pressure for them to donate their cells [35]. Gene-edited organoids with established rare genotypes can help avoid the ethical issue that arises in such cases.

CRISPR-edited patient-derived organoids hold great promise for personalized cell treatments and the replacement of impaired tissue in patients. However, CRISPR/Cas9 is known to be prone to off-target effects, which was also the case in the proof-of-concept study in organoids [13]. Off-target effects can mediate unexpected mutations at different loci, raising concerns on the safety of this genome editing technology, mainly due to its oncogenic potential. This concern is particularly relevant when organoids or cells derived from organoids are intended to be used for in vivo therapeutic applications, where genomic integrity is threatened, generating serious ethical implications. Nevertheless, continuous research has showed that off-target effects can be predicted and protocols can be refined to increase specificity of the CRISPR technology. Additionally, other Cas9 variants or other CRISPR-associated nucleases (Cpf1 and C2c1) have been shown to be highly specific and reduce off-target effects, suggesting that off-target effects will be eventually minimized. Thereupon, what is the level of safety that should be reached to allow the use of gene-edited organoids for clinical use in patients? A suggestion here is to use the existing ethical and legal framework for gene therapy clinical trials. When CRISPR is proved to achieve a safety level analogous to that of gene therapies reaching the clinical trial stage, the next step would be to study gene-edited organoids as potential therapies, in the setting of a robust, first-in-human clinical trial producing accurate evidence on safety. 

However, even in the case that an optimum level of safety has been reached for genome editing technologies, it may not be ethically acceptable to alter the human genome. Some argue that editing the human genome in cells subsequently transplanted into humans could mark the beginning of a slippery slope, which will eventually lead to other applications, being gene editing in germ cells and human embryos, human cloning or the creation of human–animal chimeras, and such applications fail to protect the fundamental value of human dignity. 

Yet again, we should consider essential differences for certain types of organoids. The special moral status attributed to embryoids and cerebral organoids and the potential use of gonadal organoids in reproduction perhaps allow their genetic manipulation and subsequent use for research purposes but not for clinical applications. A consensus should be reached between researchers on whether the use of CRISPR-edited embryoids, brain and gonadal organoids must be prohibited at the clinical level.

### 3.8. Creation of Chimeras

Transplantation of human cells in animal models and the subsequent creation of human-animal chimeras has been widely used in certain research fields. For instance, humanized mouse models are being extensively studied in cancer research, without generating massive arguments. In principle, human stem cell transplantation into animals is not distinct from transplanting human organoids into animals, but the latter may create major ethical concerns, mostly due to the fact that the transplanted human mini organs closely resemble their in vivo counterparts. 

Before all else, a key question that should be addressed in chimeric research is whether crossing species boundaries is ethically acceptable. For some, this is a violation of human dignity and human nature. Animals have a different moral and legal status from that of human beings, and are consequently treated differently. Animals are neither considered as “things” nor “persons” and do not have rights (at least yet) merely because they cannot fulfill any obligations. Quite the reverse, humans do have an obligation to protect animals and this is inherently recognized by permitting animal experimentation for scientific purposes to obtain new knowledge for the benefit of mankind, but with respect to certain principles for animal welfare, under specific legislation and under strict conditions of licensing by the competent authorities [36]. Accordingly, a primary ethical issue in chimeric research concerns animal welfare and the effects of organoid engraftment in the health of animals. In this respect, depending on the chimeric model and the human organoid used, the extent of maturation is critical and it is crucial to restrict the development of chimeric organisms, e.g., into early life instead of allowing them to reach an advanced age.

As organoid technology progresses, however, the ethical concerns grow to include particularly the use of human brain and gonadal organoids in chimeric research. When human brain organoids are transplanted in animals, this may change the cognitive capabilities of the resulting chimera [37,38]. Considering also the possibility of human brain organoids developing sentience or consciousness (as discussed earlier), such human-animal chimeras are ethically highly problematic. When human gonadal organoids are used in animals, this raises the additional possibility of cross-species fertilization. Following these possibilities for brain and gonadal organoids, the confusion as to the moral status of the chimeric organism and whether it should be treated as a human or an animal is apparent and justifiable. This is of greater concern when larger animal models than mice are used, such as non-human primates, at least due to their morphological similarity to humans.

The use of human organoids in animals probably does not require new legislation, as it falls under existing regulatory frameworks of chimeric research and animal welfare. Many European countries including Greece, Cyprus, Italy and Germany prohibit the creation of human–animal chimeras by law, mainly due to ethical issues that arise by chimeric research and the lack of ability to predict the potential outcomes of such experiments. Such prohibitions are included in existing regulations for medically assisted reproduction and in vitro fertilization. 

On the other hand, the creation of human–animal chimeras and even the development of cytoplasmic hybrid embryos for research purposes are permitted in the UK. As organoid technology progresses, legislations in the USA and Europe may need to be revised regarding chimeric research in order to avoid lagging behind in research compared to countries such as China and the UK that allow it. In any case, however, ethical scrutiny is required by the competent Research Ethics Committees reviewing the relevant protocols of organoid transplantation into animals, especially when the resulting chimeric organisms are expected to create confusion over their human or animal nature. Research protocols of human–animal chimeras involving use of human whole organoids should be reviewed on a case-by-case basis, because the potential benefits and risks and the ethical concerns which vary according to the type of organoid must be taken into consideration in each case. It is also important that research involving transplantation of human organoids into animals should be conducted gradually, closely monitoring any changes in the body and behavior of the resulting chimeric organisms at every step. 

### 3.9. Organoid Transplantation

It has been proposed that organoid technology may serve as a source of organs for transplantation, even though most researchers believe this goal is a long way off. Moving from bench to bedside, human organoids could serve an unmet worldwide need: the shortage of grafts for transplantation for replacing damaged tissues or whole organs. Of course, for clinical translation of organoids, certain standards of size, degree of maturity, organoid functionality and safety need to be achieved first. With ongoing research and continuous improvement of organoid technology, some of these obstacles are expected to fade. In a very recent example, Liu et al. managed to scale up mini-organs by using Multi-Organoid Patterning and Fusion, a robust organoid engineering approach to assemble individual airway organoids of different sizes into upscaled, scaffold-free airway tubes with predefined shapes [39], demonstrating that the size of organoids may not be a problem for transplantations in the future. 

Hence, before moving from bench to bedside, there is a need to analyze and consider the ethical issues of a first-in-human organoid transplantation trial. Such issues are not new, in the sense that they are common for all first-in-human trials involving a novel therapeutic approach. A first-in-human organoid transplantation trial would pre-require extensive preclinical research in human–animal chimeras showing sufficient evidence for organ engraftment, organ functionality and safety of the transplantation should include a full assessment of potential benefits and risks, a favorable risk–benefit balance that justifies the intervention, selection of participants and appropriate informed consent procedures. A distinction in a first-in-human organoid transplantation trial is the fact that it would require the participation of vulnerable patients in Phase I, who are at late stage of disease and urgently require organ transplantation. Therefore, it contains the risk of the so-called “therapeutic misconception”, a phenomenon during which the study participants have no other available therapeutic options and believe that they will be personally benefited therapeutically by the clinical trial rather than they will help to generate knowledge and advance the science for certain diseases [40]. In view of that, the risk of therapeutic misconception must be taken into consideration during the informed consent procedure and ensure that participants in the trial fully understand the true benefits of research.

Some scientists have even raised the question about whether it is ethically acceptable to include children in a first-in-human organoid transplantation clinical trial and under which conditions. Of course, these would be children who suffer from severe conditions that predominantly affect children, such as metabolic diseases [41]. In such cases, additional ethical concerns should be considered including the principle of subsidiarity, which demands that clinical research involving children is only permissible if the clinical study cannot be performed in adults. Such first-in-children clinical trial for liver organoid transplantation could be ethically justified provided that certain guidelines are followed and various safeguards are met [41].

Finally, it is worth noting that organoid transplantation may offer an alternative to xenotransplantation [42]. Xenotransplantation is the transplantation, implantation or infusion of living cells, tissues or whole organs from animals to humans and has been examined as a possible solution to the scarcity of human organ donors, to the illegal trade of organs from living donors and the use of condemned prisoners as donors, which is permitted in some countries. It involves genetic modification of animals (e.g., pigs and non-human primates) so that their organs cause a reduced immune response when transplanted into humans, raising arguments on the welfare of the donor animals. In addition, xenotransplantation encompasses serious safety issues, because of the possibility to transmit infectious agents from animals to human recipients threatening the recipient’s health but also public health. Perhaps more importantly, transplanting animal organs into humans raises concerns over whether this changes the human nature of the recipient and whether it violates the integrity of the human species. These issues result in reduced societal acceptability of xenotransplantation. Could organoid transplantation offer a substitute approach that can diminish such ethical concerns? Indeed, organoids are derived from humans, and therefore cannot change our human nature. They can even be derived from the recipient’s cells, vanishing the risk of transmitting (animal) diseases and minimizing the risk of organ rejection by the recipient’s immune system. 

### 3.10. Commercialization of Organoids

Human tissues and cells hold great commercial significance beyond transplantation and transfusion, and this is well exhibited through their use in organoid technology and its numerous applications in disease modelling and regenerative and precision medicine. Nevertheless, commercialization of human body parts and tissues poses ethical and legal challenges arising from the main question of whether it is possible to have property rights in biological materials extracted from the human body and consequently, whether we can sell them and have a financial gain. Opponents of human body commercialization are in favor of donation in research or therapy as an act of altruism of the donors and solidarity with those in need. In this context, it should be examined whether it is ethically acceptable to commercialize organoids derived from human tissues. On the one hand, if it is not allowed to commercialize organoids, even independently of the tissue donor’s will, then the risk of halting or placing obstacles in biomedical research is apparent. On the other hand, if organoids can be “traded” as commodities, then the interests of third parties ultimately have more value that the rights of tissue donors. Of course, property and commercialization concerns do not have a basis in the case of autologous use of human organoids, where the donor and the recipient are the same person who will potentially benefit from such a procedure.

An approach to address this difficult issue is to classify human bodily material as either subject or object, but this may not reflect their true moral value. As discussed earlier, certain types of organoids may deserve special protection due to their “special” moral status. Depending on their maturation, embryoids may closely resemble human embryos, brain organoids may exhibit even a minimal state of consciousness, or multi-organoid complexes may respond to stimuli or exhibit autonomous behavior, and therefore, their moral value is certainly higher compared to, say, kidney or intestine organoids. Accordingly, embryoids, brain organoids and multi-organoid complexes may be considered closer to “subjects” than “objects”, but as with animals which are neither considered as “things” nor “persons”, human organoids could be something between “subjects” and “objects”.

Boers et al. proposed that instead of categorizing human bodily material as either subject or object, organoids should be recognized as hybrids, which are neither human nor non-human, by considering that organoids exhibit: (i) subject-like values since they can relate to the bodily integrity of donors and recipients, to the personal identity and values of donors, to the privacy of donors and they can impact the well-being of donors, and (ii) object-like values, since they constitute biotechnological artefacts, they are a technology and they can serve as instruments to achieve scientific, clinical or commercial aims. They further described a process of legitimizing the commercialization of organoids by a detachment of the instrumental and commercial value of organoids from their associations with persons and their bodies [43]. Indeed, such an approach respects both the moral value of organoids, which stems from their connection to the cell donors and the advantages for science. 

According to normative national or European documents and guidelines, the human bodily parts (including not only whole organs but also human tissues) shall not give rise to financial gain. Nevertheless, current practices in Europe and an analysis of normative documents shows that the ban on commercialization of bodily material is not as strict as it may appear at first sight. Some countries have not ratified the relevant conventions or certain European directives and that leaves room for the commercial practice of tissue procurement and transfer [44]. Looking into the future, organoid technology with its enormous potential deserves and should have a clearer regulatory framework on whether the commercialization of human organoids is legitimate or whether it should be prohibited.

### 3.11. Patentability of Organoids

A relevant point to the commercialization of organoids is whether they should be patentable. Both dilemmas derive from the demand of property or ownership of the produced organoids. Patenting is a system of intellectual property protection designed to reward inventors. Organoids derived from human cells, either embryonic or adult stem cells, are biotechnology products resulting from the application of cell and molecular biology methods to manipulate biological processes, and thus can be deemed as patentable interventions. Indeed, a number of patents have been granted for various organoids or methods to develop organoids in the USA and Japan [45]. 

On the one hand, a robust patent system is desirable to ensure funding and progress in organoid technology, to encourage research in such beneficial areas that hold great promises in disease modelling and personalized treatments. On the other hand, in some cases, patent protection for biotechnological inventions can be limited for ethical reasons. It can be argued that the principles of beneficence and justice are not served by patent systems because patents lead to increased costs for patients and National Health Systems. At least in the European patent system, certain methods or products may be prohibited if they are contrary to “ordre public” or morality.

Particularly for organoids, once more, the type of organoid produced could play a significant role on whether it is eligible for a patent or not. Due to their special moral value, embryoids and brain organoids could be excluded from patents based on the notion of morality. Brain organoids with their potential to obtain human characteristics, cognitive abilities or to develop sentience may not be patent eligible based on the general prohibition against the patenting of immoral inventions. Likewise, the patentability of embryoids can be challenged due to their potential, particularly for the European patent system. For instance, the decision of the Courts of Justice of the European Union on the case of Oliver Brüstle v Greenpeace, related to neural precursor cells and the processes for their production from embryonic stem cells and their use for therapeutic purposes [46], illustrated that patenting of interventions that require prior destruction of human embryos or their use as base material can be problematic. The Courts of Justice of the European Union subsequently considered that the patent prohibition applies to anything functionally equivalent to an embryo with the “inherent capacity of developing into a human being”, and determined that parthenotes which are produced from an unfertilized ovum do not possess that capacity and so are patent eligible [47]. This latest decision may also have implications for the patenting of human embryoids, but considering the progress in this field and that the degree of maturity in various embryoids varies, it is difficult to assess whether embryoids have the “capacity of developing into a human being”. As a result, definitions are of major importance here, too, with implications on whether organoids will be patent eligible or not.

### 3.12. The Cost of Treatments and Issues of Equity

Commercialization and patentability of organoids have major implications on the final cost of the produced treatments. Existing examples of advanced therapies have shown that stem cell therapies may be expensive [48,49], which raises serious ethical concerns over the unequal distribution of effective therapies based on wealth and socioeconomic status. Increased cost means that not all patients in need will have access to expensive personalized treatments, despite the fact that they may be life-saving. On the other hand, iPSCs are relatively easy to obtain, which means that in the future, organoids derived from iPSCs may indeed provide a more affordable option for treatment. Thus, equity is a primary concern since the potential benefits of organoid technology should be distributed evenly.

### 3.13. Misuse and Dual Use Issues of Organoids

As with most biotechnologies, organoid technology could also be used for malevolent purposes. Rinaldi and Colotti argue that organoids can be used for harmful purposes and bioterrorism. For instance, lung and brain organoids could be used to test the toxicity of new chemical weapons, toxic chemicals or toxins, or to assess the infectivity of biological agents [50]. More than other in vitro cell systems, the knowledge gained through the use of organoids can also be used for military applications. Biobots combining robots and human tissues, such as organoids, are typical examples of items raising dual-use concerns. This is particularly possible for brain organoids connected to a body, such as a robot, not necessarily a human-like robot. Small insect- or amphibian-like robots can provide a “vector” for military applications with even autonomous or semi-autonomous properties.

Such malevolent and dual use applications must be considered at an early stage because, as the technology progresses, certain characteristics or “abilities” of organoids evolve. Raising these ethical issues among researchers is a necessary first step to prevent such applications. Although researchers have benign intentions when they develop and experiment on organoid technology and its applications, this does not mean that the technology or the knowledge gained by it cannot fall in the wrong hands. Special regulations may not be necessary for dual and malevolent use of organoids, but current legislations and ethics standards covering the potential misuse or dual use of biotechnologies can be applicable in organoids, too.

### 3.14. Organoid Biobanking

Organoid biobanking is extremely important for translational research. Organoid biobanks constitute living biobanks storing viable cells, tissues or even whole mini organs that can play a double role in research (e.g., alternatives for drug testing in animals, disease modeling, models of organ development) and clinical settings (e.g., precision medicine, regenerative medicine, transplantation). Large collections of different types of organoids representing the genetic heterogeneity of healthy individuals or patients with various diseases offer tremendous advantages for the study of human diseases and the development of treatments. 

Small or larger collections of patient-derived organoids have already been established, mainly for cancer studies. These biobanks store patient-derived tumor and matching healthy organoids including colorectal cancer, pancreatic ductal adenocarcinoma, breast cancer, prostate cancer and liver cancer organoids, mainly used to test drug sensitivity (reviewed in [51]). Recently, the first pediatric cancer organoid biobank containing tumor and matching normal kidney organoids was also set up, aiming to capture the heterogeneity of pediatric kidney tumors [52]. The potential advantages of organoid technologies have led large, international initiatives, such as the Human Cancer Models Initiative (HCMI) [53], to join forces and generate large biobanks of organoids available for the research community.

Nevertheless, organoid biobanking has ethical implications. Some concerns are old but new ethical issues arise due to the very nature of organoids. At the current stage of organoid biobanking, there are no binding rules, principles or legal norms defining the rights and duties of donors and biobankers. Notably, the ambiguous moral and legal status of organoids further complicates the issue of who owns the cell-derived organoids. Organoids are biological entities that do not clearly fall into the categories of cells, gametes, tissues or organs which are legally regulated under relevant laws. Defining the legal status of organoids, including certain types such as brain organoids, gonadal organoids and embryoids, is the cornerstone for the consent of cell donors and the subsequent uses of organoids (e.g., research, clinical, not-for profit, for-profit). Ultimately, defining the legal status of organoids is a central element in the governance of organoid biobanks.

Commercialization of organoid biobanks raises the issue of fairness and can affect the donors’ trust and their willingness to provide their samples [54]. Anonymization of the samples would practically make organoids ownerless but this approach does not allow donors to maintain their right to withdraw consent. The ownership status of organoids becomes even more ambiguous if organoids are modified through gene editing, which means that the final organoid has been produced by means of a technical process, allowing room for patenting. To overcome the issue of ownership, many existing biobanks that store and use human biological materials have agreed to be custodians or trustees. A similar strategy can also be applicable to organoids biobanks. Custodians can act as the organization that actually holds the assets and trustees can act as managers of the assets for the beneficiaries of a trust or other party. The literature also suggests that the idea of treating participants more like “partners” rather than passive tissue “donors” makes biobank governance more ethically responsible and fair, particularly in the context of living organoids derived from stem cells of donors [54].

Organoid biobanking demands proper informed consent strategies for both research and clinical purposes. Similarly to biobanking of human cells and tissues, different consent approaches can be followed in organoid biobanking: (a) a blanket consent without any limitations, (b) a broad consent with some restrictions, (c) a tiered consent for certain areas (e.g., cancer), or for specific diseases (e.g., breast cancer), or (d) a continuous consent, which requires re-consent for new uses or purposes. There is no consensus on the most suitable type of consent for organoid biobanking. As in many other cases, a continuous consent would be impractical and requires an investment of time and resources that impedes the accomplishment of biobanks’ aims. On the one hand, the more specific a consent is, the more control is given to donors over their donation. On the other hand, in order to prevent losing potential social benefits from the use of organoids, a broad consent may be a better option in organoid biobanking, as long as donors are provided with sufficient information to make a reasonably informed decision.

However, donors may have specific concerns, as in the case that the biobank is commercial or for-profit. Therefore, a significant point which must not be missed in the informed consent is whether the cell donor is informed about the prospect of commercialization of organoids, and whether he/she agrees to it. Objections may also arise based on the type of organoid that is biobanked. For brain organoids or embryoids, donors may feel more attached to them compared to other organoids. Opt-out options should be available in such cases, providing donors the opportunity to object to certain uses or purposes (e.g., object to use after the donor’s death, non-therapeutic uses, commercial purposes), according to their personal values and beliefs. In any case, the consent procedure is and should remain central in the governance of organoids biobanks, to ensure voluntary and well-informed donation of samples.

In biobanking, donors provide their consent (whether broad or specific) based on the condition that their privacy and personal data are protected by de-identification of the samples. Perhaps one of the major harm risks in biobanking is associated with breaking privacy of donors. One approach to de-identification is anonymization of samples. This may be applicable for organoid biobanks for research purposes only, in which case the return of results to donors may not be necessary. Nevertheless, one should not overlook the skepticism that true anonymization of genetic data may not be feasible, due to their very nature. Some believe that the availability of DNA sequencing technology can make it difficult to maintain anonymization without previous agreements to not pursue identification via next-generation sequencing.

However, for evident reasons, anonymization is unsuitable for biobanks that eventually use organoids for clinical applications, such as personalized treatments, precision medicine and transplantation, as none of these therapeutic approaches are feasible without knowing the donor’s identity. The decision of patients to donate their stem cells for organoid biobanking partly depends on the possibility of them being cured from severe diseases for which no other effective treatments exist. Thus, anonymization is not deemed appropriate in this case.

What we also need to take into consideration is the fact that organoids are accompanied by genetic data, which are sensitive personal data and demand robust measures of data protection, particularly for organoids stored long-term and used many years after the original stem cell donation. Again, this issue must be addressed during the informed consent procedure. At the same time, bankers and investigators are legally and ethically obligated to protect sensitive data of donors. They are required to take appropriate measures to minimize the risk of unauthorized third parties obtaining access to health and genetic data. Finally, the unclear legal status of organoids and the ambiguous ownership status also have implications on the ownership of the genotypic or phenotypic data produced in organoid studies, and this deserves close consideration.

When some of these organoid applications move from research to clinical uses, e.g., the production of personalized treatments, further considerations must be taken into account. The clinical validation of organoids must precede and subsequently, possible risks and benefits of the treatment, of alternative treatments and of refusing the treatment must also be considered. As a matter of fact, every research activity or clinical application does involve a certain level of risk for participants or patients. As with human cell and tissue biobanking, a key issue is that the potential risks to cells donors are disproportionate to the overall benefits of organoids biobanking. Obviously, this does not lift the obligation of bankers and researchers to take every possible measure to protect donors from such risks. In any case, the long-term storage and use of “live” organoids demands meticulous, continuous ethics review and oversight by independent ethics bodies. Members of these Ethics Bodies should have a high level of expertise in ethics, law, organoid technology and biobanking, and of course, representatives of donors or patients should also participate.

Finally, let us not forget the lessons learnt from the past regarding human biological material biobanks. What will the fate of organoids be upon unexpected or planned closure of a biobank? In this respect, a strategy must be in place in each biobank to handle the organoids according to the relevant legislation but also according to the donor’s informed consent. This, of course, requires that the possibility of closure of the biobank has been taken into consideration during the informed consent procedure. Instead of losing the benefits from previous work on organoids, perhaps the best plan in case of closure is to ensure that stored organoids are preserved by transferring the biobank’s resources to another entity [55]. In addition, the organization level of organoid biobanks will play a key role in their sustainability, but also in the quality of services provided. To protect and ensure a high quality of research and services, organoid biobanks should implement standard operating procedures, quality assurance and quality control programs. In order to achieve consistency in their practices, organoid biobanks should also obtain accreditation, which requires previous dedication of staff and resources. This is particularly important as organoid biobanking is expected to increase in the near to mid-term future.

## 4. Concluding Remarks

Organoid technology holds great promises as alternatives for animal experiments, disease modeling, regenerative medicine, precision medicine and transplantation. However, this technology raises complex ethical issues related to the moral and legal status of organoids, informed consent and privacy of donors, property rights and governance of biobanks, in both research and clinical settings. A special moral status can be attributed to certain types of organoids, such as brain and gonadal organoids, creating debates amongst scientists and members of society on whether they demand special protection compared to other organoids. In the present manuscript, the ethical challenges posed by organoid technology have been analyzed and specific recommendations on ethical and regulatory oversight have been offered.

In view of the fact that up to this moment, there are no specific regulations or guidelines for organoid use in research and clinical care, a general combined approach should be followed to achieve ethical use of organoid technology. The first step would be to examine if existing ethics review processes, guidelines and regulatory frameworks are also applicable to organoids. To the degree that organoids show similarities with hESCs or iPSCs, their use can be examined through existing guidelines of the International Society for Stem Cell Research (ISSCR) for both stem cell research and clinical translation [56], which could be adapted if necessary. For organoids used for the development of novel therapies, the standard approaches to ethics oversight in gene therapy and the relevant legislation may also be applicable. Likewise, for long term storage of organoids in biobanks, existing oversight mechanisms in human biological material and DNA biobanking could be extended to ensure ethically sound strategies for organoid biobanking.

Even so, some of the ethical challenges posed by organoids are not specifically addressed. Therefore, a second step is required to ensure ethical use of organoids. This is to examine whether specific types of organoids or specific applications demand special regulatory provisions. This certainly includes the case of brain organoids and embryoids, which may have an increased moral status. For instance, existing legislations in various jurisdictions regulating in vitro fertilization and embryo research may not be appropriate for embryoids that are not a product of egg fertilization. In such cases, specific regulatory frameworks will promote and support ethical organoid research or applications in clinical care.

A third complementary step would be essential to ensure societal acceptance of organoid use and participation in relevant research. This is to engage the public and promote a dialogue between science and civil society on the ethical issues around organoids including informed consent and privacy, and experimenting with human brain tissues and embryo-like tissues. Public engagement will also help minimize public confusion and misinterpretations of using “mini-organs in a dish” and at the same time will avoid promises of organoid technology that cannot be confirmed. Of course, this needs to be combined with appropriate public (media) communication to avoid hyperboles and excessive expectations of organoid use.

A final, equally important step to ensure ethical oversight and ethical use of organoids would be to continuously monitor the rapid advancements of this technology. This is particularly important as organoid research moves into clinical trials to ensure that any new ethics issues or any changes in the complexity of existing issues will be taken into consideration. 

This four-step approach will help maximize the biomedical and social benefits of organoid technology. Despite the multifaceted and complex ethical challenges posed by organoid use and biobanking, we have a moral obligation to make sure that we do not lose any of the potential benefits through careful considerations, ethical and legal oversight.
